# Enhancing ultrasound education in obstetrics and gynecology in Germany: insights and innovations from postgraduate training

**DOI:** 10.1007/s00404-024-07610-2

**Published:** 2024-06-26

**Authors:** Marie-Sovie Schlapp, Agnes Wittek, Ricarda Neubauer, Annegret Geipel, Ulrich Gembruch, Brigitte Strizek, Florian Recker

**Affiliations:** Department of Obstetrics and Prenatal Medicine, University Hospital Bonn, Venusberg Campus 1, 53127 Bonn, Germany

**Keywords:** Ultrasound, Training, Education, Postgraduate education, Residency, Curriculum development

## Abstract

**Background:**

Ultrasound (US) has become integral to obstetrics and gynecology (Ob/Gyn), necessitating proficient training during residency. Despite its clinical importance, there is a perceived gap in the quality and structure of postgraduate ultrasound education in Germany.

**Methods:**

A cross-sectional survey was conducted among Ob/Gyn residents in Germany from October 2023 to March 2024, using the LimeSurvey platform. The survey, distributed via email, social media, and physical flyers, garnered 211 participants, with 115 completing all sections. The questionnaire covered demographic details, US training experiences, and the perceived importance of US in clinical practice.

**Results:**

Although US was highly valued by respondents, with an average of 26.1% of their clinical activity devoted to it, there was significant dissatisfaction with the training quality. Key issues included inadequate supervision, the necessity of self-training, and low participation in certification courses. Despite 93.0% awareness of professional US organizations like the German Society for Ultrasound in Medicine (DEGUM), engagement with structured training programs was minimal.

**Discussion:**

The study highlights a critical need for standardized US training protocols and curricular reform in Ob/Gyn residencies in Germany. The discrepancy between the recognized importance of US and the quality of training indicates a pressing need for improvements. Addressing these gaps through comprehensive, structured educational reforms could significantly enhance the proficiency and confidence of future Ob/Gyn specialists, ultimately improving patient care.

## What does this study add to the clinical work

**Table:** 

This study sheds light on the gap between the clinical importance of US and the adequacy of postgraduate training in obstetrics and gynecology, underscoring a critical need for standardized training frameworks. It reveals a significant reliance on self-learning among residents, highlighting the urgency for structured supervision and mentorship. The research identifies barriers within hospital operations that hinder comprehensive US training, advocating for organizational reforms to support structured education. It is of significant international importance, offering insights that transcend national boundaries. By highlighting a gap in postgraduate training quality and structure, it sheds light on a universal challenge within the medical education sector. The research advocates for standardized training frameworks and comprehensive educational reforms, proposing a model that could be adapted and implemented across various healthcare systems globally. This initiative aims to align postgraduate education with clinical demands, ultimately improving patient care. The findings and recommendations presented could inspire similar studies and innovations worldwide, fostering international collaboration and exchange of best practices in ultrasound education. Ultimately, this study emphasizes the need for proficient and confident specialists in obstetrics and gynecology, contributing to enhanced diagnostics, treatment, and healthcare outcomes on a global scale.	

## Introduction

Ultrasound (US) technology, with its profound impact on clinical diagnostics and therapeutic interventions, has become an indispensable tool in the area of obstetrics and gynecology (Ob/Gyn). This technology offers a non-invasive, real-time visualization of fetal development, maternal structures, and aids in the diagnosis and management of various obstetrical and gynecological conditions. Traditionally, the acquisition of ultrasound skills was a journey embarked upon during the postgraduate phase, more specifically, throughout the residency programs dedicated to aspiring gynecologists and obstetricians [1]. However, the dynamic nature of medical education and the increasing complexity of patient care have prompted a reevaluation of how and when these crucial US skills are taught and mastered [2].

The incorporation of ultrasound in medical education (USMed) signifies a pivotal shift, emphasizing the importance of early exposure and the systematic development of US competency [3]. This approach not only prepares future physicians with a foundational skill set but also aligns with the evolving demands of patient care in Ob/Gyn [4], where US plays a critical role in monitoring fetal health, guiding interventional procedures, and managing obstetric emergencies [5].

Germany has been at the forefront of integrating USMed into its medical curriculum, a move catalyzed by the introduction of the National Competency Based Catalog of Learning Objectives for Undergraduate Medical Education (NKLM) in 2015 [6]. This strategic inclusion mandates that medical students develop the ability to employ US as an adjunct to traditional clinical examinations, setting a precedent for its application in specialized fields such as Ob/Gyn [7, 8].

Despite the structured approach to USMed at the undergraduate level, the pathway to US proficiency during residency in Ob/Gyn remains heterogeneous. This variability spans from informal, experiential learning to formalized training programs, highlighting a gap in standardized education which is potentially affecting the quality of patient care [9].

Internationally, there is no single standard for US education in OB/GYN residency programs, leading to considerable variability in how training is structured and delivered. Some countries, such as the United States and those in the European Union, have developed comprehensive guidelines and curricula that outline the expected competencies and minimum training requirements [10]. For instance, the American Institute of Ultrasound in Medicine (AIUM) [5] and the European Board and College of Obstetrics and Gynecology (EBCOG) and International Society of Ultrasound in Obstetrics and Gynecology (ISUOG) offer detailed recommendations for US training [11–13].

Recognizing the indispensable role of US in Ob/Gyn, this manuscript delves into the existing frameworks of postgraduate US education for residents in these specialties [14]. It aims to map out the educational landscape, identify the methodologies employed in teaching US, and uncover the challenges and barriers faced by residents in acquiring and refining their US skills.

Furthermore, this study will explore the perceptions of residents regarding their US training, including their confidence in performing US examinations, the adequacy of their educational experiences, as well as the impact of their training on clinical proficiency and patient outcomes. Through an in-depth analysis, we seek to uncover the disparities in US education across different residency programs and to highlight the need for a more uniform and comprehensive approach.

By examining the current state of US education in postgraduate training for Ob/Gyn residents, this manuscript aims to contribute valuable insights into how US training can be optimized. The goal is to ensure that emerging specialists are not only proficient in the technical aspects of US but are also capable of integrating this knowledge into patient-centered care. In doing so, we aspire to lay the groundwork for future advancements in US education, ultimately enhancing the quality of healthcare delivery in the field of Ob/Gyn.

## Methods

### Questionnaire and distribution

A comprehensive, voluntary and anonymous online survey was designed to capture insight in the perspectives and opinions of residents regarding their US training in the field of Ob/Gyn. The detailed questionnaire can be found in Attachment 1 for further reference. The cross-sectional needs assessment survey was made available for participation over a 6 month period, starting from October 2023 until March 2024. To facilitate the distribution and collection of responses, the study utilized the LimeSurvey platform (LimeSurvey, Version 6.4.2) [15], known for its robust data collection and analysis capabilities.

To maintain the integrity of the survey results, we implemented IP address tracking to prevent multiple submissions from a single participant, thereby ensuring the validity and reliability of the collected data. It is important to note that participants were not offered any form of financial incentive to take part in the study, emphasizing the voluntary nature of their contribution.

An introductory passage on the LimeSurvey platform, delineating the objectives of the survey and detailing its collaborative initiative with the representation for residents from the German Society of Gynecology and Obstetrics, constituted the initial engagement with potential respondents. This introductory text not only extended an invitation for participation but also offered an exhaustive elucidation of the survey’s importance and the anticipatory roles of the participants.

The strategy employed for the propagation of this study was multifaceted, including both primary and secondary dissemination channels. Primarily, electronic mail communication was utilized to reach residents and various educational entities recognized for their contributions to US training programs. Additionally, a secondary strategy involved the physical distribution of flyers equipped with QR codes, which directed to the online questionnaire. These flyers were strategically placed in numerous national healthcare facilities, practices, and disseminated during medical training events.

Beyond these direct outreach efforts, social media platforms, notably Facebook and Instagram, were harnessed to expand the survey’s reach, employing posts and reminders in specific groups to boost participation rates and ensure a broad spectrum of responses.

The survey itself was segmented into three discrete sections, encompassing a total of 32 queries aimed at garnering a holistic perspective on the residents’ experiences and perspectives regarding US training in the area of Ob/Gyn. The preliminary section of the questionnaire was designed to collate demographic data and background specifics of the respondents, including their affiliations with healthcare institutions and their stage of postgraduate education. The ensuing segment was dedicated to eliciting detailed insights into the US training, incorporating a self-assessment and evaluation predominantly via a 5-point Likert scale. The final section focused on various independent educational US institutions and concluded with a series of final inquiries.

This study was performed in line with the principles of the Declaration of Helsinki. Approval was granted by the Ethics Committee of University Bonn (180/23-EP).

### Statistics

Raw data extracted from the online survey were downloaded as a Microsoft Excel® Spreadsheet for preliminary organization. For the statistical analyses, Microsoft Excel®(Microsoft Office® LTSC Professional Plus 2021 Version 2108) was used with counting formulas and corresponding diagrams were within created. For each survey question, the results were displayed as absolute numbers and in percentages (%). The percentages were rounded to one decimal place.

## Results

In this study, a cohort of 211 participants engaged in the survey, with 115 residents (54.5%) completing the questionnaire in its entirety, abstaining from omitting any items. Eligibility for participation was contingent upon current enrollment in a German Ob/Gyn Residency Program at the time of this investigation. Despite concerted efforts, it was challenging to secure fully completed questionnaires from residents employed in practices. This phenomenon may be attributable to the regulatory framework in Germany, which permits only a single year of practice-based experience to be recognized for accreditation, leading a majority of residents to undertake their entire training within hospital settings.

The study achieved the completion of 115 questionnaires from residents across Germany. In the absence of an official registry detailing the number of Ob/Gyn residents, membership figures from the Young Forum (Junges Forum, JF) of the German Society for Gynecology and Obstetrics (Deutsche Gesellschaft für Gynäkologie und Geburtshilfe, DGGG) were employed as a proxy indicator. The Young Forum reported approximately 2500 members as of October 2023, the inception of this survey. Accordingly, the response rate to the survey among German Ob/Gyn residents was calculated to be 4.6%, based on the completion of the questionnaires by members of the Ob/Gyn Program.

### Demographic characteristics

Participants across all levels of postgraduate training contributed to the completion of the questionnaire. A notable proportion, 24.3% (28 participants), were in their 5th year or beyond, corresponding to the final year of residency training in Germany (Table 1). The demographic composition revealed that the majority, 90.4%, of respondents were aged between 25 and 34 years. Gender distribution among the resident respondents was significantly skewed, with 92.2% (106 out of 115) identifying as female, while the remaining 7.8% (9 participants) identified as male [14]. In terms of employment setting, a plurality of the respondents reported currently being employed at a university hospital, accounting for 36.5% (42 participants) of the total, followed closely by those working at maximum care providers, representing 33.9% (39 participants) of the responses.

**Table 1:** Demographic characteristics of survey respondents: this table outlines the demographic profile of the residents who participated in the survey, including their postgraduate year level, age, gender, and type of hospital where they are currently employed. The data is presented in terms of the number of participants and their corresponding percentage of the total respondent pool, providing insights into the diverse backgrounds of the residents engaged in US training in Ob/Gyn

Characteristics	Number of participants (n) Percentage (%)
Resident postgraduate level:	
1 year	20 (17.4)
2 year	27 (23.5)
3 year	23 (20.0)
4 year	17 (14.8)
5 year or more	28 (24.3)
Age:	
20—24 years	0 (0.0)
25—29 years	53 (46.1)
30—34 years	51 (44.3)
35—39 years	10 (8.7)
40—45 years	1 (0.9)
Gender:	
Female	106 (92.2)
Male	9 (7.8)
Diverse	0 (0.0)
Type of hospital:	
University hospital	42 (36.5)
Maximum care provider	39 (33.9)
Regional hospital	33 (28.7)
Practice	0 (0.0)
Other	1 (0.9)
Total	115 (100.0)

Among the respondents, 62.6% (*n* = 72) envisioned continuing their professional endeavors within a hospital setting following the completion of their residency. Meanwhile, 23.5% (*n* = 27) anticipated engaging in practice-based work, and 10.4% (*n* = 12) aimed to pursue careers in ambulatory healthcare centers (Medizinische Versorgungszentren, MVZ). A minority of 1.7% (*n* = 2) indicated “other” options, specifying uncertainty regarding their future professional trajectories.

### Residents experience in US training and perceived importance of Ob/Gyn and prenatal US

Most responding residents estimated to spend approximately 5 hours per week with gynecological (63/54.8%) and obstetric/prenatal (60/52.2%) diagnostical US (Table 2).

**Table 2:** Weekly hours dedicated to diagnostic US: this table presents the estimated hours per week that residents dedicated to performing diagnostic US in both gynecological and obstetrical/prenatal contexts. It categorizes the responses into various time intervals, illustrating the distribution of weekly time commitment to US diagnostics among the residents

	Gynecological US	Obstetrical/prenatal US
0 h/week	11 (9.6%)	13 (11.3%)
approx. 5 h/week	63 (54.8%)	60 (52.2%)
approx. 10 h/week	32 (27.8%)	30 (26.1%)
approx. 20 h/week	6 (5.2%)	11 (9.6%)
more than 20 h/week	3 (2.6%)	1 (0.9%)

No participants rated the level of supervision during their residency as “very good”. A fraction of respondents, 15.7% (*n* = 18), considered it “good”, while 36.5% (*n* = 42) found it to be moderate, neither good nor bad. A significant proportion, 40.0% (*n* = 46), deemed the supervision “bad” and 7.8% (*n* = 9) classified it as “very bad”.

**Fig. 1:**
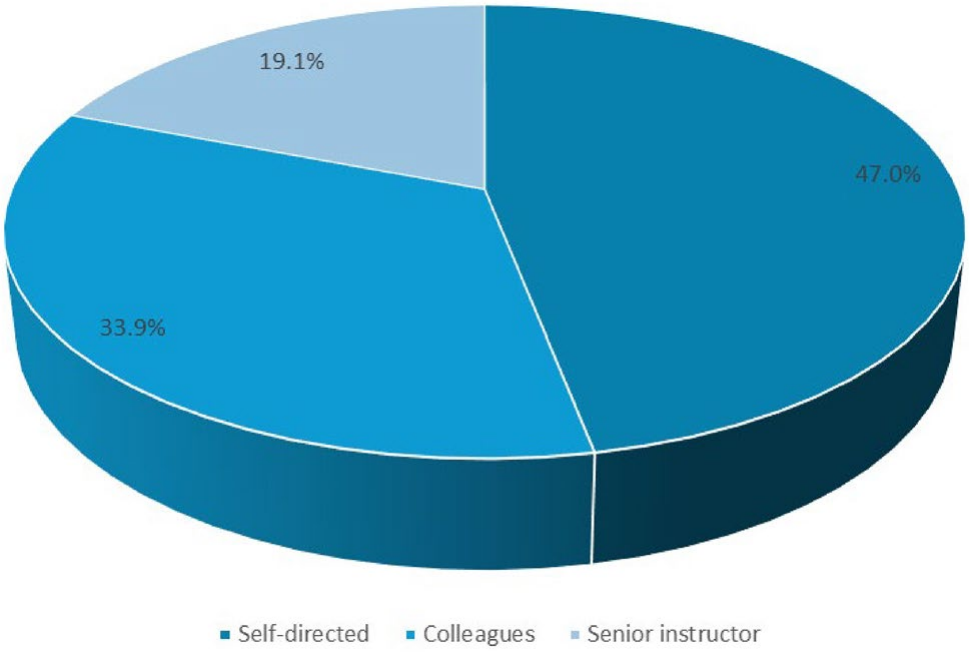
Distribution of ultrasound trainers: this figure shows who trains the residents in ultrasound

Regarding training modalities, 47.0% (*n* = 54) of the residents reported self-directed learning as their primary method of training. Additionally, 33.9% (*n* = 39) received training from their colleagues, and 19.1% (*n* = 22) were instructed by senior physicians (Fig. 1).

The survey participants reported that US constitutes approximately 26.1% of their clinical activities. In terms of the perceived importance of sonography in daily clinical operations, 48.7% (*n* = 56) of the respondents attributed a very high significance to US, 41.7% (*n* = 48) considered it high, 7.8% (*n* = 9) deemed it medium, 0.9% (*n* = 1) rated it as low, and another 0.9% (*n* = 1) as very low.

**Fig. 2:**
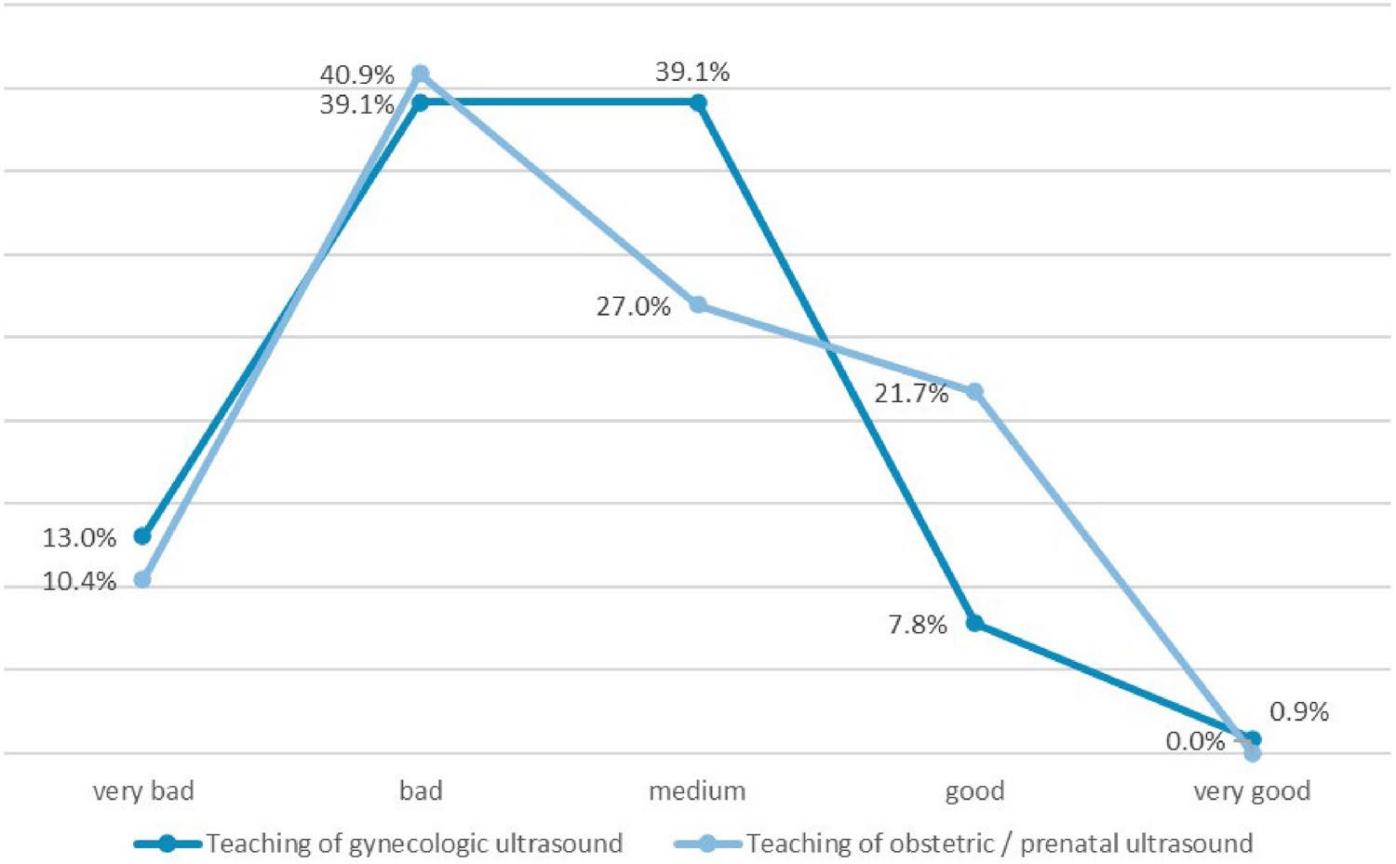
Evaluation of ultrasound teaching: this figure represents the assessment of gynecologic and obstetrical/prenatal ultrasound teaching

Evaluation of US teaching yielded that 39.1% (*n* = 45) of the residents rated the instruction in gynecological US as predominantly bad, and another 39.1% (*n* = 45) as moderate, and only 7.8% as good. Dissatisfaction with obstetric and prenatal US teaching was expressed by 40.9% (*n* = 47) of the residents (Fig. 2).

Further, despite 90.4% (*n* = 104) of respondents recognizing the high to very high significance of sonography in their daily clinical practice, there exists a notable gap in structured postgraduate US education. Specifically, 52.1% (*n* = 60) and 51.3% (*n* = 59) expressed dissatisfaction with the quality of gynecological and obstetric/prenatal US training.

The frequency of residents summoning their trainers for patient consultations per week was distributed as follows: 7.8% (*n* = 9) very rarely/never, 30.4% (*n* = 35) rarely, 29.6% (*n* = 34) occasionally, 27.0% (*n* = 31) often, and 5.2% (*n* = 6) very often.

Interestingly, 27.0% (*n* = 31) of residents frequently sought assistance from their US trainers for patient examinations weekly, yet 48.4% (*n* = 15) of this group also stated that they have to train themselves. Among the 30.4% (*n* = 35) who seldom involved their US trainers in patient examinations, 51.4% (*n* = 18) of them indicated they resorted to self-training.

Concerning the competency in making sonographic diagnoses in routine clinical practice, participants expressed feeling overwhelmed with varying frequencies: 4.3% (*n* = 5) very often, 28.7% (*n* = 33) often, 35.7% (*n* = 41) occasionally, 30.4% (*n* = 35) rarely, and 0.9% (*n* = 1) very rarely/never. The sole respondent who never felt overwhelmed was in her fifth postgraduate year, affiliated with a university, invested 1 h per week in training, and noted the necessity of self-directed learning.

Residents dedicated an average of 1.2 h weekly to sonographic training, primarily utilizing US textbooks/literature (57.4%, *n* = 66), US courses (49.6%, *n* = 57), and seeking advice from designated supervisors within their institutions (46.1%, *n* = 53). Additionally, 8.7% (*n* = 10) employed alternative methods such as online courses and job shadowing, while the same percentage did not engage in sonographic training at all.

Regarding the development of obstetric/prenatal US skills since the onset of residency, respondents classified their progress as good (46.1%, *n* = 53) or average (35.7%, *n* = 41), with a minority rating it poorly (bad 8.7%, *n* = 10 and very bad 4.3%, *n* = 5). Similar trends were observed in the advancement of gynecological US skills, with the majority assessing their development as average (44.3%, *n* = 51) or good (36.5%, *n* = 42).

**Fig. 3:**
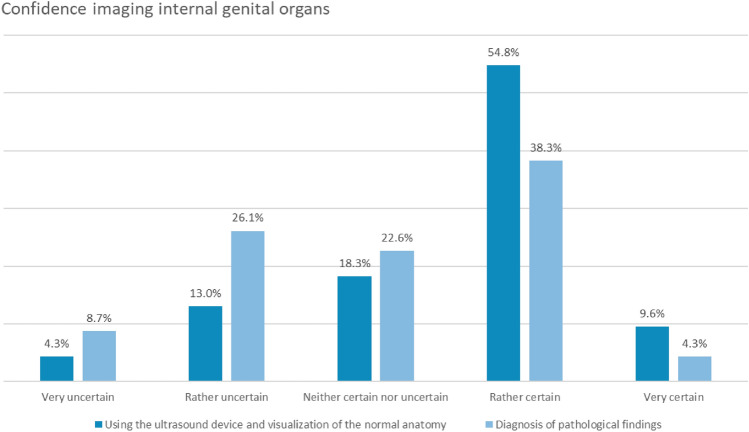
This figure illustrates the feeling of confidence of the residents while imaging the normal and pathological internal genital organs

**Fig. 4:**
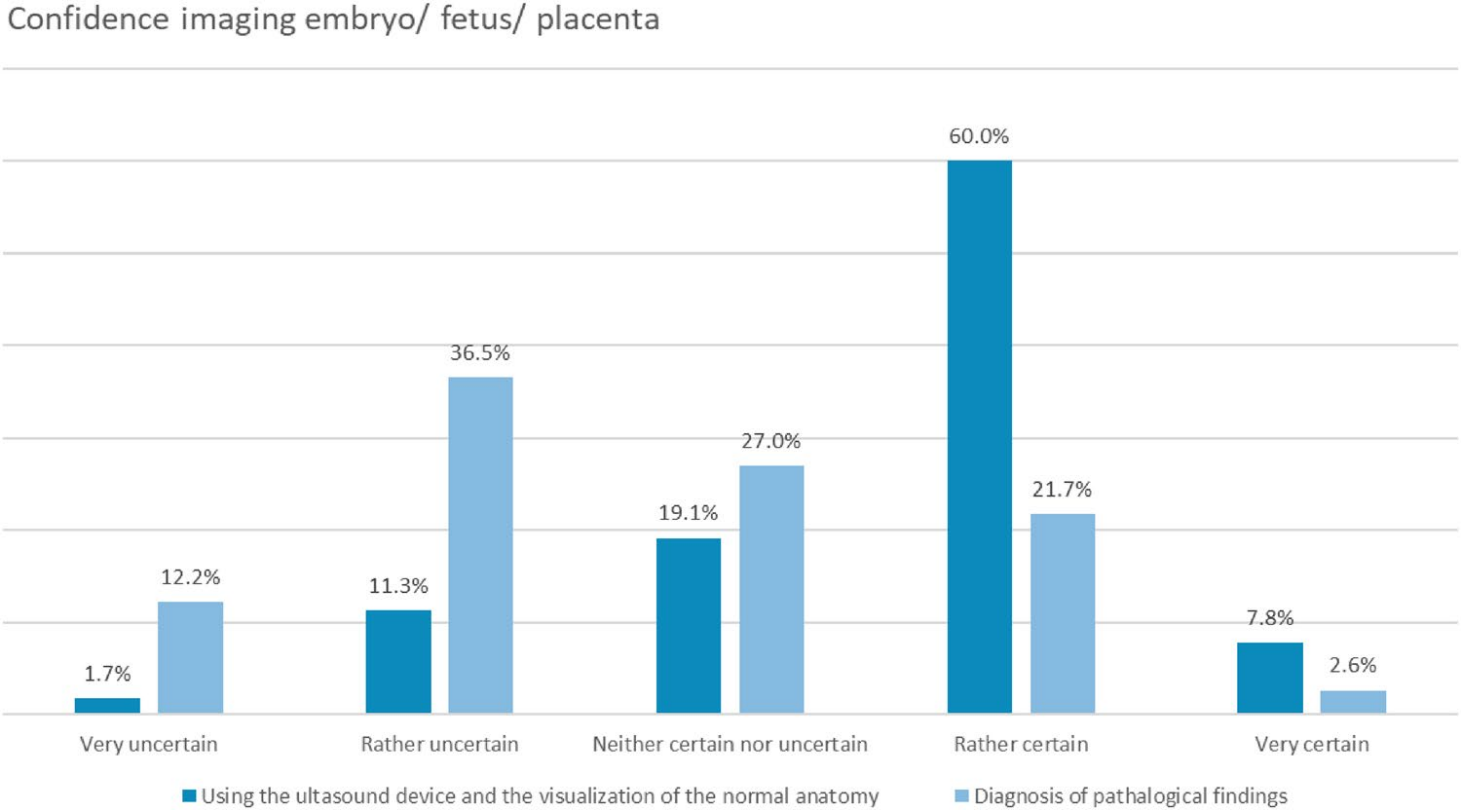
This figure depicts the feeling of confidence of residents during imaging the normal and pathological embryo/fetus/placenta

Confidence in utilizing US equipment and identifying normal sonographic anatomy of the female internal genitalia, as well as embryonic, fetal or placental structures, was reported to be high among a significant proportion of the residents. However, there was a notable degree of uncertainty regarding the diagnosis of pathological conditions within these domains (Figs. 3, 4).

Although a major proportion of respondents frequently felt overwhelmed by the responsibility of making sonographic diagnoses in everyday clinical settings with 4.3% (*n* = 5) very often and 28.7% (*n* = 33) often, the confidence was higher in diagnosing normal findings, uncertainty increased when identifying pathological conditions.

### Information regarding US organizations and postliminary questions

A significant proportion of the survey participants, 93.0% (*n* = 107), reported familiarity with the German Society for Ultrasound in Medicine (DEGUM). However, a majority, 80.9% (*n* = 93), had not attended any International Ovarian Tumor Analysis (IOTA) certification courses, and 83.5% (*n* = 96) had not participated in courses offered by the Fetal Medicine Foundation (FMF). Furthermore, 42.6% (*n* = 49) of the residents were employed at institutions recognized as DEGUM training centers, while 20.0% (*n* = 23) were uncertain about their workplace’s status as a DEGUM training center.

Regarding the certification levels of their instructors, 37.4% (*n* = 43) held DEGUM Level II certification, 20.9% (*n* = 24) possessed DEGUM Level III, and 19.1% (*n* = 22) had no DEGUM certification, 11.3% (*n* = 13) of the instructors had a DEGUM Level I. Additionally, the same amount (*n* = 13) of respondents were unaware of their instructor’s DEGUM certification status.

A striking 87.0% (*n* = 100) of respondents indicated the absence of structured, regular teaching within their clinical routines. A significant majority, 67.0% (*n* = 77), perceived US examinations as being underrepresented in the new training catalog. Moreover, 79.1% (*n* = 91) of residents expressed concerns about their preparedness for potential sonographic responsibilities in practice settings due to the current training regimen.

The majority, 56.5% (*n* = 65), felt that the structure of hospital operations impedes the organization of structured US training, highlighting a perceived gap in the training framework necessary to meet the demands of future sonographic activities in clinical practice.

## Discussion

The findings of this survey underscore a crucial gap in the postgraduate US education landscape for Ob/Gyn residents in Germany. Despite the recognized importance of US in clinical practice, a significant disparity exists between the perceived value of sonographic skills and the structured training provided during residency. The study’s results illuminate several critical areas for improvement and potential pathways forward.

The overwhelming majority of residents acknowledges the critical role of US in their daily clinical activities, with nearly half attributing a very high importance to it. This recognition underscores the necessity for comprehensive and structured US training programs that are not currently being met, as evidenced by the considerable percentage of residents who reported dissatisfaction with the quality of both gynecological and obstetric/prenatal US training. The dissatisfaction is further compounded by the lack of regular, structured teaching, with a significant 87% of respondents indicating its absence in their clinical routine. An American survey of program directors in Ob/Gyn also revealed the same data here [5].

The issue of inadequate supervision and the need for self-directed learning strategies highlighted by nearly half of the survey participants raises concerns about the consistency and quality of US education during residency [15]. Moreover, the reliance on self-training and informal learning from peers, rather than structured guidance from certified instructors, may contribute to the variability in skill acquisition and confidence among residents [17].

This finding suggests a pressing need for a more formalized and standardized approach to US training, including clearly defined competencies, benchmarks for proficiency, and regular assessment of skills [16, 17].

With the Project for Achieving Consensus in Training (PACT), the European Board and College of Obstetrics and Gynecology (EBCOG) defined common objectives for training in Ob/Gyn residency programs providing the groundwork for higher curricular standardization on European level [11]. Besides specifying concrete US skills to achieve, the importance of US training itself is highlighted by a separate chapter emphasizing a systematic step-by-step approach. In accordance with the recommendations of the International Society of Ultrasound in Obstetrics and Gynecology (ISUOG) Education Committee, US training should be progressing in a structured manner from learning the theoretical basics to supervised application in a clinical context and finalizing with a proof of competence through direct observation, the documentation of a critical number of US examinations and images as well as formal assessments [11, 13].

The respondents’ sense of being overwhelmed by sonographic diagnoses in clinical practice as well as the low self-perceived confidence in identifying pathologic conditions (see Figs. 3, 4), further emphasizes the importance of not only technical training in US but also in the development of interpretative and decision-making skills [12]. This aspect of training is crucial for ensuring that residents are well-prepared to integrate US findings into patient care effectively and confidently.

Interestingly, the study revealed a substantial awareness among residents for the DEGUM as the respective national US society, yet a low participation rate in certification courses offered by DEGUM, IOTA, and the FMF. This gap between awareness and engagement with professional US organizations suggests potential barriers to access, such as time constraints, lack of awareness of the benefits, or perceived relevance of these certifications to their future careers. Addressing these barriers by integrating such certifications into residency training programs could enhance the quality and standardization of US education.

The perception that US examinations are underrepresented in the new training catalog and the widespread concern about preparedness for sonographic responsibilities in future practice settings underscore the need for curricular reform [18]. In recent years, consensus recommendations regarding the content and structure of US training have created a common basis for the implementation of standardized US curricula [11, 13]. Nevertheless, the findings of this study suggest that existing guidelines are yet not sufficiently implemented in actual clinical training programs. A structural reform embedding comprehensive US training within the residency curriculum, aligned with national and international standards on US core competencies and training delivery, could bridge the current gap and better prepare residents for the demands of clinical practice [9]. Furthermore, in addition to the systematic integration of US education into daily practice, structural barriers need to be addressed that currently inhibit the take up of advanced training offers from specialist societies.

Finally, the structural challenges within hospital operations that impede the organization of structured US training point to a broader issue within the healthcare system. Addressing these challenges requires a multifaceted approach, including administrative support, resource allocation, and perhaps most importantly, a cultural shift towards valuing and prioritizing US education as an integral component of residency training.

### Limitations

One of the primary limitations of this study is its reliance on self-reported data, which inherently carries the risk of subjective bias. Participants’ assessments of their US training experiences, perceived competence, and the importance of US in their clinical practice might be influenced by individual perceptions and memory recall, potentially skewing the results. Additionally, the voluntary nature of the survey could lead to a selection bias, with more motivated or concerned residents being more likely to participate, which may not accurately represent the broader population of obstetrics and gynecology residents in Germany.

The study also did not differentiate between the specific content, e.g. obstetrical ultrasound for antenatal surveillance, fetal anomaly scanning, reproductive medicine, senology, and the quality of US training in general received by the residents. This lack of granularity means that the findings provide a broad overview rather than an in-depth analysis of specific educational interventions or their effectiveness. Consequently, it is challenging to identify which aspects of US training are most in need of improvement or are most beneficial.

Furthermore, the study’s cross-sectional design limits the ability to infer causality or track changes in US training quality and residents’ confidence over time. A longitudinal approach would provide a more dynamic view of how US training impacts residents’ competencies and confidence throughout their residency and into their professional careers.

## Conclusion

In conclusion, this study highlights a critical need for reform in postgraduate US education for Ob/Gyn residents. By addressing the identified gaps and barriers, there is an opportunity to enhance the quality of care provided to patients and to better prepare residents for the increasingly important role of US in gynecological and obstetrical medicine. Future directions should focus on the development of standardized curricula, increased access to certification and training programs, and systemic changes within hospital operations to support structured, comprehensive US education.

## Data Availability

Data are available upon request from the authors.
